# Digital Forensic Research for Analyzing Drone Pilot: Focusing on DJI Remote Controller

**DOI:** 10.3390/s23218934

**Published:** 2023-11-02

**Authors:** Sungwon Lee, Hyeongmin Seo, Dohyun Kim

**Affiliations:** 1 Department of Computer Engineering, Catholic University of Pusan, Busan 46252, Republic of Korea; glick925@naver.com; 2 The Affiliated Institute of Electronics and Telecommunications Research Institute (ETRI), Daejeon 34044, Republic of Korea; sylar@nsr.re.kr; 3 Department of Computer and Information Engineering, Catholic University of Pusan, Busan 46252, Republic of Korea

**Keywords:** drone, remote controller, DJI RC, drone forensics, UAV forensics

## Abstract

Drones, also known as unmanned aerial vehicles (UAVs) and sometimes referred to as ‘Mobile IoT’ or ‘Flying IoT’, are widely adopted worldwide, with their market share continuously increasing. While drones are generally harnessed for a wide range of positive applications, recent instances of drones being employed as lethal weapons in conflicts between countries like Russia, Ukraine, Israel, Palestine, and Hamas have demonstrated the potential consequences of their misuse. Such misuse poses a significant threat to cybersecurity and human lives, thereby highlighting the need for research to swiftly and accurately analyze drone-related crimes, identify the responsible pilot, and establish when and what illegal actions were carried out. In contrast to existing research, involving limited data collection and analysis of the drone, our study focused on collecting and rigorously analyzing data without restrictions from the remote controller used to operate the drone. This comprehensive approach allowed us to unveil essential details, including the pilot’s account information, the specific drone used, pairing timestamps, the pilot’s operational location, the drone’s flight path, and the content captured during flights. We developed methodologies and proposed artifacts to reveal these specifics, which were supported by real-world data. Significantly, this study is the pioneering digital forensic investigation of remote controller devices. We meticulously collected and analyzed all internal data, and we even employed reverse engineering to decrypt critical information files. These achievements hold substantial significance. The outcomes of this research are expected to serve as a digital forensic methodology for drone systems, thereby making valuable contributions to numerous investigations.

## 1. Introduction

With the recent advancements in IoT technology, unmanned aerial vehicles (UAVs), particularly drones, have become widely utilized worldwide. These small wireless embedded devices, equipped with wireless communication technology, are connected to controllers and capable of taking to the skies, thus earning them the moniker ‘Mobile IoT’ or ‘Flying IoT’. Drones find utility in various domains, including law enforcement and public safety operations [1,2], search and rescue missions [3], and agriculture [4]. As of 2023, the drone market has generated a revenue of $3.99 billion. It is expected to witness an 8.3% growth in sales by 2024 and is projected to continue growing at an average annual rate of 3.24% until 2028. Global drone sales are estimated to reach a staggering 9.27 million units [5].

However, it is essential to acknowledge that drones are not always used for positive purposes. Due to their small size and ability for remote control, drones can access hard-to-reach places where humans cannot go. As a result, they have been utilized in recent military conflicts and wars, and the potential for their misuse in criminal activities raises serious concerns.

For example, drones have been widely and aggressively used for lethal purposes in conflicts such as the Russia–Ukraine conflict [6], the Israel–Palestine conflict [7], and the Israel–Hamas conflict [8]. Modern warfare is sometimes referred to as ‘drone warfare’. Additionally, in December 2022, it was revealed that five North Korean drones had crossed the inter-Korean border and conducted reconnaissance flights over Seoul’s airspace [9]. Despite attempts by the South Korean military to shoot down these drones using helicopters and jet fighters, they were unsuccessful, thus leading to comparisons of the situation to ’shooting a fly with a cannonball’.

Drones’ small size and remote control capabilities make them challenging to track, and it is often difficult to identify the operators [10]. For these reasons, since the late 2010s, a growing body of digital forensic research has focused on preparing for the potential misuse of drones in various criminal activities.

Most existing research predominantly revolves around collecting and analyzing data from DJI drones and smartphones used as panels in remote controllers. Researchers often rely on the DJI’s provided software, Assistant, for data collection to extract encrypted drone flight records. The analysis of these encrypted files usually depends on third-party programs such as CsvView/DatCon [11], AirData UAV [12], and Phantom Help [13]. This means that researchers are limited in their ability to access files directly from the device’s storage, thus resulting in a restricted scope of collectible data. Additionally, the analysis of the collected encrypted files is often tied to the functionalities of these third-party programs.

Furthermore, while smartphones were primarily used as panels for drone control in the past, recent developments have led to the creation and widespread use of remote controllers with their dedicated interfaces. Unfortunately, there is a lack of digital forensic research dedicated to these modern remote controllers, which now incorporate their own integrated panels.

Drones come in various types and a wide range of quantities, and manufactourers are continuously releasing new products. This means that conducting digital forensic research on each new drone model requires significant time, effort, and resources. In contrast, remote controllers, which are essential for drone flight, have a lower frequency of new product releases. They typically offer compatibility with most of the manufacturer’s drone models. Furthermore, remote controllers often run more complex operating systems like Android than the simple real-time operating systems (RTOS) commonly found in drones. This complexity enables various functionalities beyond essential drone flight control, including features like account management, firmware updates for the drone and the controller, and more.

For these reasons, we believe that remote controllers are likely to store crucial data, such as pilot account information, drone connections, and flight details. Hence, we conducted digital forensic research on remote controllers to explore these potential sources of valuable information.

We chose DJI’s remote controller, DJI RC, as the focus of our research, given DJI’s dominant position with a 76% market share in the global drone market [14,15]. DJI RC stands out, as it includes an independent panel, thereby eliminating the need for a separate smartphone for drone operation. Its embedded Android operating system can wirelessly control, monitor, and manage drone operations. This design allows DJI RC to store information that is crucial for digital forensics investigations, including details about the drone pilot, paired drones, flight records, and captured media. Consequently, DJI RC can serve as valuable evidence in digital forensic inquiries.

Our research aimed to collect and analyze all the data from DJI RC. Ultimately, we aimed to uncover critical information, including who was operating the drone, when they connected to which drone, the pilot’s location during flight, the drone’s flight path, and any potentially illegal activities conducted during drone operations. To achieve this goal, we researched methods to collect data freely from all files within the flash memory of DJI RC, rather than limiting our data collection as in previous research. We then meticulously analyzed all system information, installed apps, system logs, app logs, and more within DJI RC. In this process, we reverse engineered the installed apps to decrypt any encrypted data. As a result, we extracted critical artifacts that are crucial from a digital forensics perspective to answer the seven Ws (WHO, WHICH, WHEN, WHERE, WHERE to WHERE, WHAT) for pilot investigation:

- Q1: Who operated the drone (WHO)?
- Q2: Which drone was operated (WHICH)?
- Q3: When was the drone operated (WHEN)?
- Q4: Where was the drone operated (WHERE)?
- Q5: From where to where was the drone’s flight controlled (WHERE to WHERE)?
- Q6: What did the drone capture (WHAT)?

The contributions of this research can be summarized as follows:

- **Pioneering Digital Forensics for DJI RC:** In a field lacking digital forensics research on RC devices, this study proposes a comprehensive approach for data collection and analysis specific to DJI RC.
- **Entire Data Acquisition and In-Depth Analysis:** Unlike prior research, the research focuses on the complete collection of data from DJI RC, thereby conducting detailed analysis to identify crucial artifacts necessary for achieving the objectives of digital forensics investigations.
- **The Decryption of DJI’s Unique Encryption Methods:** The study introduces methods for decrypting files that contain vital information encrypted and encoded using DJI’s proprietary techniques.

These contributions significantly advance the digital forensics field, particularly in drone-related investigations, thus offering innovative methodologies and techniques for digital forensics researchers and investigators.

This paper consists of a total of eight chapters. Section 2 introduces prior digital forensics research related to drones. Section 3 explains the methodology employed for conducting this research. Section 4 provides an overview of DJI’s drone remote controller (RC), thereby highlighting its features and various information. Section 5 explores methods for collecting data from DJI RC’s internal flash memory and external SD cards. Section 6 delves into the analysis of the collected data, including file system analysis, app behavior analysis, and artifact analysis. Section 7 summarizes the research findings and discusses their implications. Section 8 concludes the research and outlines directions for future work.

## 2. Related Work

The research on drones from a cybersecurity perspective has been underway since the early 2010s. Various vulnerability assessments and studies aimed at enhancing their security against hacking were initially conducted. During that period, the research primarily focused on products from manufacturers such as China’s DJI and France’s Parrot, as they dominated the drone market. In the latter part of the 2010s, digital forensic research was initiated to investigate drone involvement in criminal activities. Since DJI continued to hold a significant share of the global drone market during this period [14,15], most studies focused on DJI products. We reviewed 11 papers published since 2017, as summarized in Table 1. These papers cover the types of devices analyzed, the data collection areas and methods, the types of data analyzed, and the tools used for analysis.

The core focus of digital forensic investigations on drones is to determine who—with which specific drone and remote controller—operated the drone, where it flew from and to, and the activities conducted during flight, especially in criminal use cases. To achieve these objectives, most existing studies have primarily concentrated on analyzing the data from within the drones [16,17,18,19,20,21,22,23,24,25,26]. Among these, six studies have also includes the analysis of the remote controller. However, it is worth noting that this controller is essentially a smartphone used for piloting the drone through a dedicated app rather than a unique device developed by the drone manufacturer [17,18,19,20,23,25]. Thus, research analyzing dedicated drone remote controller devices provided by drone manufacturers has yet to be undertaken.

Early remote controllers released by drone manufacturers were equipped with small real-time operating systems (RTOS) and featured only basic joystick controls for piloting. Users had to connect their smartphones to the remote controller to enable functions like real-time drone control screens and various communication features. These remote controllers offered the advantage of affordability, but they had the drawback of depending on the user’s smartphone for all drone control functions. Recently, remote controllers have evolved to include dedicated panels for drone piloting, such as DJI’s DJI RC, which facilitates seamless integration with various drones released by DJI. These advanced controllers are equipped with more complex operating systems, like Android, and host multiple dedicated apps for drone flight, thereby ensuring a more stable and reliable drone control experience. Consequently, many users opt for these devices. This shift towards more advanced remote controllers necessitates digital forensic research, and our study has chosen DJI’s DJI RC as the subject of investigation.

Most previous studies have relied on the DJI Assistant program provided by DJI for data collection. Drone pilots commonly use this tool to extract flight records from their drone devices, thereby allowing them to review their flight logs on a PC. This tool extracts the drone’s flight records in an encrypted DAT file format, thus making it inaccessible to users in its raw form. However, third-party programs like CsvView/DatCon [11] can be employed for data decryption and flight log visualization, thus enabling users to access and analyze flight records.

However, collecting data through the DJI Assistant program has the disadvantage of being akin to extracting iPhone data into backup files via iTunes, where investigators have limited control over the selection of data required for their investigation. Therefore, for a thorough digital forensic examination, there is a need for research that accesses the device’s storage at the file system level to either image all the data or extract the individual files necessary for analysis. While one study among the existing research imaged the flash memory of a drone to extract all the data, it used a chip-off method and, therefore, cannot be considered a universal data collection technique [21]. Additionally, some prior studies rooted the Motorola G3 and Samsung Galaxy S7 smartphones used as panels for remote controllers and imaged their flash memories [17,23]. However, this method is not universally applicable, as the rooting process varies depending on the smartphone model, thereby making it impractical as a generalized data collection method.

However, in our research, we developed methods for collecting data from the DJI RC that involved rooting the device, imaging the entire flash memory, and selectively extracting encrypted data through the shell. As a result, we were able to gather a significantly more considerable amount of data for digital forensics compared to existing research. Furthermore, in contrast to previous research being constrained by data collection limitations, we conducted a comprehensive analysis by examining all the files within the file system of the DJI RC. We went beyond this by identifying all installed apps on the device, thus conducting a thorough analysis of their behavior through reverse engineering of the installation files and delving into the analysis of the encryption algorithms for critical files. As a result, we extracted important artifacts for digital forensics by analyzing logs from the overall system and the installed applications. This approach provided a more extensive and in-depth understanding of the digital forensics, thus surpassing the focus on DAT files and SD card data that has been prevalent in earlier research.

The following is a brief review of the content of previous research.

Devon R et al. [16] researched data collected from DJI’s Phantom III drone. They focused on extracting and parsing critical data and successfully decrypting encrypted information. They also developed a drone data analysis tool called Drone Open Source Parser (DROP). They utilized DROP to analyze DAT files that stored the DJI Phantom III flight records and investigated correlations among the log files. It is worth noting that, although they reverse-engineered the CsvView/DatCon program to decrypt encrypted data, the encryption method for DAT files has since changed, thereby rendering their approach obsolete.

Barton et al. [17] focused on data collection and analysis for DJI’s Phantom 3 Professional, Parrot’s A.R. Drone 2.0, two different drones, and an Android-based Motorola G3 smartphone used as a remote controller for drones. They developed open-source tools for this purpose. They created analysis methods based on virtual crime scenarios, thereby exploring the analysis of drone flight records and traces of interactions with the primary devices.

Maryam et al. [18] conducted data collection and analysis for DJI’s Mavic Air drone and data from an iPhone 6 used as the DJI Mavic Air remote controller panel. They utilized DJI Assistant for collecting drone data, and for analyzing the DAT files containing the drone’s flight records. They employed the CsvView/DatCon tool [11]. Other data sources were exclusively from SD cards, and in the case of the iPhone 6, they collected and analyzed the backup data using iTunes.

Ankit et al. [19] researched Yuneec Typhoon H and DJI Phantom 4 drones, as well as an Android smartphone used as the panel for the remote controller. They proposed a forensic framework for drone data collection and analysis. Both drones were connected via USB to collect the data, and the flight logs were analyzed using Airdata UAV. They extracted TXT files containing drone flight logs from the SD card on the smartphone and analyzed the flight records using the Phantom Help service.

In addition to their research in 2019, Maryam et al. [20] expanded their investigations in 2020 to include DJI Mavic 2 Pro, Mavic Air, Spart, and Phantom 4 drones. They employed similar data collection methods using DJI Assistant and iTunes, and the utilized the FTK Imager for collecting data from SD cards. For data analysis, they employed various tools, including FTK Imager, Autopsy, Encase, Prodiscover Basic, and Paraben E3: Universal, and they further analyzed the DAT files containing drone flight records using CsvView/DatCon and EtractDJI.

Fahad E et al. [21] conducted a research study on two types of drones, DJI Matrice 210 and Phantom 4, where the flash memory data was collected through the chip-off method. The collected data were then analyzed using digital forensic tools, including Autopsy, Cellebrite Physical Analyzer, AXIOM, and CsvView/DatCon, with a focus on drone flight record analysis. Notably, this research was significant, as it represented the first study to collect all of the flash memory data from the drones using the chip-off method, thereby eliminating the need for DJI Assistant in the data collection process.

Fahad E et al. [22] conducted research involving small drones like the VTI Phoenix and medium-sized drones like the DJI Matrice 210. The VTI Phoenix utilized Nmap scanning to identify open ports on the device and successfully executed a brute force attack on the port 21 (FTP) to gain root access to the shell. In the case of the DJI Matrice 210, they followed the conventional approach by using DJI Assistant to extract the DAT files containing the drone’s flight records, and they analyzed these files using CsvView/DatCon.

Miloš et al. [23] conducted digital forensics on the DJI Mini 2, DJI RC-N1, and two smartphones used as panels (the iPhone 7 and Samsung Galaxy S7). They executed five different scenarios and then proceeded with data collection and analysis. The collected files included the DAT and TXT files that stored the drone flight records. The DAT files extracted from the drone were encrypted, and to decrypt them, they used the forensic tool CsvView/DatCon to extract the KML, GPX, and CSV files contained within. The extracted CSV files were also encrypted, and they used the airdata.com web service for decryption. This process enabled them to analyze the drone’s flight records. They also extracted the TXT files containing the drone flight records from both smartphone types and used the forensic tool Cellebrite to extract the JPEG files within these TXT files. By analyzing the EXIF data, they determined the latitude, longitude, and timestamps when the photos were taken.

Lee et al. [25] analyzed three types of drones—DJI Phantom 3, DJI Mavic Mini, and DJI Mini 2—on two smartphones—the iPhone 7 and Samsung Galaxy S7—used as panels for the remote controller. They classified the DJI drone products based on the encryption method of the extracted flight logs and analyzed the multimedia data from SD cards. They proposed a method for smartphones to identify the DJI models by analyzing the multimedia data captured by the drones.

In addition to direct data collection and analysis on drone devices, some researchers have conducted studies where they collected and analyzed drone data from known open datasets. Hudan et al. [24] analyzed data from the SD cards of seven DJI drone models, connected to Android and iOS devices, and they presented a methodology for timeline analysis. They also researched the development of forensic tools. Vikas et al. [26] classified and reviewed various attack techniques applicable to drones in papers published between 2018 and 2023. These attack techniques targeted drones, communication networks, base stations, remote controllers, and authentication, and they categorized the artifacts that can be acquired through these attacks. They also compiled a list of forensic tools that can be used to analyze these artifacts. Additionally, they outlined security models applicable to drones for anti-forensic techniques and listed drone datasets. Furthermore, they summarized a drone forensic framework.

## 3. Methodology

A comprehensive understanding of the DJI RC is essential to achieving our research objectives. Based on this understanding, various functions of the DJI RC, such as pairing with the drone and logging into the DJI servers, need to be executed, with a focus on generating user data through drone flights, photo and video captures, multimedia file transfers, and other activities.

Subsequently, when considering the hardware and software features of the DJI RC, a method was established for collecting and analyzing the data from the DJI RC. Finally, based on the analyzed content, we aimed to uncover traces related to criminal activities through a seven Ws analysis, which involved examining five categories of artifacts: pilot information, information on drones paired with the DJI RC, pilot location information during drone flights, drone location information for flight records, and information regarding the multimedia data captured by the drone.

### 3.1. A Comprehensive Understanding of DJI RC

We conducted a detailed analysis of the hardware and software configuration of DJI RC, along with the connection information with other systems, such as PCs and DJI servers. Understanding this information is crucial for performing various actions to leave maximum user traces on DJI RC. Additionally, from a digital forensics perspective, considering the hardware and software characteristics is essential when collecting data from embedded devices. Therefore, in Section 4, we thoroughly examined the hardware and software composition of DJI RC and the data exchanges between connected systems to facilitate a comprehensive understanding of the device and its interactions with the surrounding environment. This analysis is a foundation for our research, thereby enabling us to conduct practical digital forensics on embedded devices like DJI RC.

### 3.2. User Trace Creation on DJI RC

From a digital forensics perspective, various user traces must be stored on DJI RC to derive artifact information for the seven Ws analysis. Initially, we paired DJI RC with the drone, connected it to a wireless network, and accessed DJI servers. This process involves binding user accounts, DJI RC, and the drone, thereby preparing the drone for flight. Subsequently, DJI RC was used to fly the drone multiple times, thus capturing photos and videos during flights and transmitting the generated multimedia files back to DJI RC through different scenarios.

The traces generated by all these user actions have been thoroughly analyzed in Section 5. This analysis aimed to extract artifact information related to user activities, thereby allowing for a comprehensive understanding of the data stored on DJI RC from a digital forensics perspective.

### 3.3. DJI RC Data Acquisition from a Digital Forensics Perspective

The method for collecting digital forensics data varies depending on the type and characteristics of the digital device. For instance, with PCs, the hard drive can be physically separated from the motherboard, thereby enabling hardware-based digital forensics tools to connect to this storage device. This allows for imaging internal data or selective collection of crime-related files.

In contrast, embedded devices like IoT typically use a system-on-chip (SoC) architecture, where the flash memory containing data is soldered onto the motherboard. In such cases, physical separation of the flash memory through a technique known as ’chip-off’ is possible, followed by data collection using a memory reader. However, this process can increase the risk of damaging the device, so the software-based approach is more common for data collection. DJI RC falls into the category of embedded devices, much like IoT devices. It contains a Linux kernel and the Android operating system internally, thus making it possible to use digital forensics data collection methods similar to those used for Android smartphones. Detailed information on this topic is explained in Section 5.

### 3.4. DJI RC Data Analysis to Digital Forensic Investigation

We conducted a detailed analysis of how all the traces we have generated have been stored, including whether any user traces were encrypted, and we ensured that nothing was overlooked. During this process, we extracted APK files, which are the installation files for all the apps installed on DJI RC. Through reverse engineering of these APK files, we uncovered the behavior of each app to identify and analyze user traces comprehensively.

After analyzing all individual files containing user information, we categorized the artifacts required for 7 Ws analysis into five categories: pilot information, information about drones paired with DJI RC, pilot location information during drone flights, drone location information for flight records, and information regarding multimedia data captured by the drone. These details are elaborated upon in Section 6.

## 4. DJI Drone Remote Controller

We selected the DJI RC, which can control most drones released using the DJI, as the subject of our analysis. Detailed specifications for this device are provided in Table 2. In this chapter, we initially verified the basic information about this device and analyzed its features.

The DJI RC is a controller with a panel that runs on a customized Android OS 10 with a Linux kernel. Users can control drones through the DJI Fly app installed on the Android OS. An unusual aspect of this OS is that it forces the DJI Fly app to run persistently in the foreground, thereby preventing users from navigating to the Android home screen, launching other apps, or closing the DJI Fly app. Consequently, users can only use the DJI RC for drone control via the DJI Fly app and not for other purposes like web browsing.

Furthermore, this OS does not allow the installation of third-party apps, thus making it impossible to install apps via external SD cards. Additionally, it restricts access to external URLs and does not permit the download and installation of third-party apps, even though the embedded WebView is in the DJI Fly app.

Regarding firmware updates for the DJI RC, there are two methods available. The first involves connecting the DJI RC to Wi-Fi and performing firmware updates through the DJI Fly app. The second method entails connecting the DJI RC to a PC via a USB serial port and downloading the DJI Assistant 2 application from the official DJI website for firmware updates.

### 4.1. Connection with Peripherals

**Figure 1:**
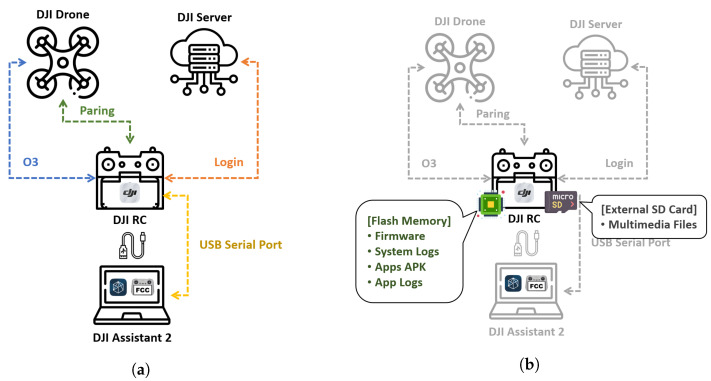
Information of DJI RC. (**a**) Connection information of DJI RC. (**b**) Data to digital forensic analysis of DJI RC.

The DJI RC can establish connections with various devices, as illustrated in Figure 1a. Here is the information regarding the connections and communication methods among these devices:

- *DJI RC and DJI Drone*
  The DJI RC pairs with the DJI Drone through the DJI Fly app. Notably, during pairing attempts, the DJI Fly app always verifies the drone’s firmware version and permits pairing only if it is up to date. Once pairing is complete, users can control the drone’s flight using the DJI RC’s sticks. The real-time flight feed transmitted from the drone’s camera is also sent to the DJI RC via the Ocusync 3.0 protocol.
- *DJI RC and DJI Server*
  In order to fly the drone normally, it is necessary to register an account and log in to the DJI server through the DJI Fly app on the DJI RC. Once logged in, the DJI Server checks if the firmware version of the connected drone is up to date and restricts the drone’s flight range based on the permissible altitude and distance set by each country. If the DJI RC is used to fly the drone without logging in to the DJI Server, the flight altitude is limited to 30 m, and the distance is capped at 50 m, thus making regular drone operation impossible.
- *DJI RC and PC with DJI Assistant 2*
  The DJI RC can be connected to a PC via a USB cable. By installing the DJI Assistant 2 application on the PC and installing the device drivers for the DJI RC, the PC recognizes the DJI RC as an external storage device and allows USB serial communication. Afterward, users can access the SD card inside the DJI RC through File Explorer to view photos and videos captured by the drone and update the device’s firmware.
  Furthermore, if the DJI RC has been rooted, it becomes possible to establish shell communication between the DJI RC and the PC via the ADB protocol. Details regarding rooting the DJI RC are elaborated in Section 5.1.1.

The DJI RC exhibits some unique characteristics regarding its connection with the drone. When the DJI RC is connected to a PC via USB and communication is established through the ADB protocol, it becomes impossible to pair the DJI RC with the drone—furthermore, attempting to establish a connection via the ADB protocol between the PC and the DJI RC while controlling the drone’s flight with the DJI RC results in the disconnection of the pairing between the DJI RC and the drone. Therefore, when the DJI RC is being used to control the drone’s flight, it is impossible to analyze the DJI Fly app dynamically.

### 4.2. Data to Digital Forensic Analysis

The data targeted for analysis on the DJI RC is stored across two physical storage media, as depicted in Figure 1b: internal flash memory and an external SD card. The internal flash memory contains several partitions, including the firmware files for the DJI RC, the system logs for the Android OS, the installation and execution files for installed apps, and the log files generated by these apps. All of these components are subject to analysis.

The data on the external SD card can be collected and analyzed without the need for any special administrator privileges. This can be achieved by extracting the micro SD card from the DJI RC and using a card reader for data retrieval and subsequent analysis. This storage location contains photos and video files captured by the drone, which are typically downloaded by the DJI RC from the drone during its operations. The DJI RC typically stores multimedia files captured by the drone on the external SD card. Analyzing these files enables the examination of photos and videos taken by the drone, and, particularly, analyzing the EXIF data within photo files allows for the analysis of aspects such as the drone’s flight path.

## 5. Data Acquisition

We researched methods for collecting data generated when operating drones using the DJI RC and DJI RC-N1. As previously mentioned, the DJI RC is a device equipped with an independent, customized Android OS. Therefore, we can utilize the data collection methods that are commonly used for Android smart devices to collect data from it.

### 5.1. Internal Flash Memory

As depicted in Figure 1b, the data on the DJI RC exist on two storage media: the internal flash memory and the external SD card. To access and collect data from the internal flash memory, standard data collection methods used for Android smartphones can be applied, as referenced in previous studies [27,28,29]. Acquiring root privileges on the device’s kernel is essential to accomplish this. Therefore, we utilized the DJI RC FCC program for this purpose [30].

#### 5.1.1. Rooting

The DJI RC FCC tool is a hacking utility designed to allow users to modify the transmission power level mode of the DJI’s remote controllers, specifically the DJI RC and DJI RC PRO, by switching between CE/FCC modes. The CE mode is a mode set for drone flights in European countries. It has weaker signal strength, which limits the drone’s range and makes it susceptible to interference. In contrast, the FCC mode is configured for drone flights in regions such as the United States, Canada, and China. It offers a more excellent range for drone flights compared to the CE mode and is more resilient against unwanted signal loss.

Therefore, in countries where the DJI RC is set to CE mode for drone flights, the DJI RC FCC tool can be used to change the configuration to FCC mode. This modification allows users to operate the drone safely at greater distances beyond the restricted areas, thereby effectively extending the drone’s range during flights.

**Figure 2:**
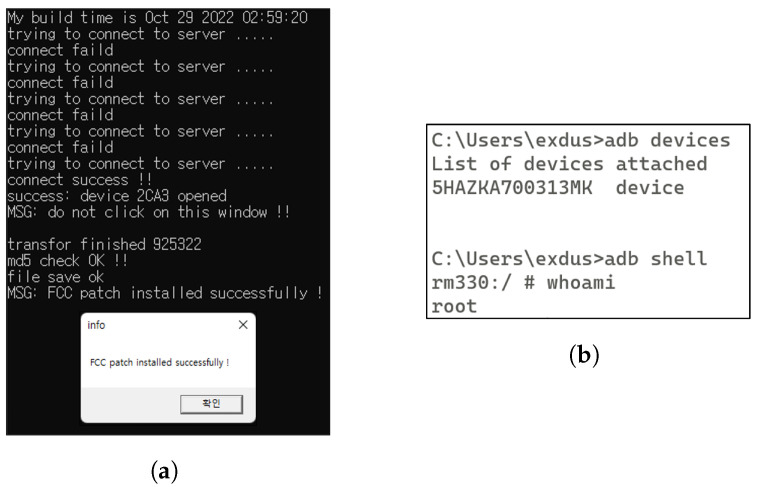
DJI RC rooting using DJI RC FCC. (**a**) Successful rooting using DJI RC FCC. (**b**) Check root privilege using ADB shell.

The DJI RC FCC tool utilizes an exploit to gain root privileges on the DJI RC during the mode-changing process. To root our research target, the DJI RC, we used version V01.03.0100 of this tool. The rooting process involves the following steps:

1. Initially, we launched DJI Assistant 2 and connected the DJI RC to the PC via USB.
2. We then installed the DJI RC’s device driver on the PC.
3. Next, we ran the DJI RC FCC tool. Following a few reboots of the DJI RC, the rooting process was completed, as indicated by the message “FCC patch installed successfully!” shown in Figure 2.
4. Subsequently, we attempted to establish a shell connection to the DJI RC from the PC using the ADB protocol. This allowed us to confirm that root privileges had been successfully and permanently secured on the DJI RC.

This rooting process gave us elevated access and control over the DJI RC, thereby facilitating further research and data collection.

#### 5.1.2. Flash Memory Imaging

**Figure 3:**
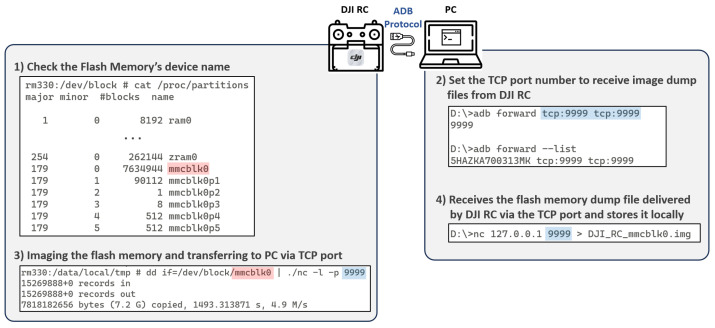
Imaging procedure of the DJI RC flash memory.

Having obtained root privileges on the DJI RC through the DJI RC FCC, we could utilize shell access to image the internal flash memory of the DJI RC. The internal flash memory of the DJI RC has a capacity of 8GB, and it is partitioned into a total of 67 partitions using the GUID partition table (GPT), as shown in Table 3. To collect all partitions of the flash memory, we followed the four steps outlined below, as depicted in Figure 3, to image the mmcblk0 block device:

1. First, using the command cat/proc/partitions, we confirmed the block device name, mmcblk0, which represents the entire flash memory.
2. Next, to extract the imaging dump file from the DJI RC to the PC, we used the adb forward command to specify a port number (e.g., 9999) for the ADB TCP protocol.
3. Simultaneously, on the DJI RC, we used the dd command to image the mmcblk0 block device, and we used the netcat program to transmit it to the PC over port 9999.
4. On the PC, we received the transmitted image dump file from the DJI RC using the netcat program over port 9999.

These steps enabled us to image the flash memory of the DJI RC and transfer the dump file to the PC for further analysis.

#### 5.1.3. Extracting the Encrypted Files

In the /data partition of the DJI RC, crucial digital forensic data is stored, including the device information, installed app files, app logs, and the internal SD card. However, as indicated in Table 4, there are encrypted directories and files within the subpaths of the /data partition. These encrypted data components pose a challenge for digital forensics because they are collected in their encrypted state when imaging the flash memory using the previously mentioned method. Consequently, additional decryption processes are required to access and analyze this data.

However, once root privileges are obtained on the DJI RC, accessing the /data partition through the shell provides access to these data components in their decrypted state. Consequently, the files that are encrypted and the focus of digital forensic analysis need to be additionally extracted using the adb pull [source] [destination] command in the ADB shell. The paths to the data that need to be extracted using this method are as follows:

- /data/app/*;
- /data/app-lib/*;
- /data/misc/*;
- /data/system/*.

### 5.2. External SD Card

The DJI RC external SD card can be imaged after physically removing it and using hardware imaging equipment. Our research used MediaImager’s GM4 Pro [31] for imaging the external SD card. If the hardware imaging equipment is unavailable, it is possible to image the external SD card by inserting it into a write blocker and connecting it to a PC. Forensic software tools such as EnCase [32] or FTK Imager [33] can be used for imaging in such cases.

## 6. Data Analysis

This chapter analyzes the data collected from the internal flash memory and external SD card of the DJI RC. Firstly, we analyzes the file system information of each storage medium to gain an overview of the internal data. Following that, we reverse engineered the behavior of the apps installed on the DJI RC, and, subsequently, we analyzed the log data generated by each app. Finally, we analyzed the multimedia data captured by the drone.

The goal of this analysis was to identify the artifacts necessary to answer the seven Ws, which are the objectives of our digital forensic investigation:

- Q1: Who operated the drone (WHO)?
- Q2: Which drone was operated (WHICH)?
- Q3: When was the drone operated (WHEN)?
- Q4: Where was the drone operated (WHERE)?
- Q5: From where to where was the drone’s flight controlled (WHERE to WHERE)?
- Q6: What did the drone capture (WHAT)?

We aimed to achieve the objectives of our digital forensic investigation by analyzing these artifacts and data.

### 6.1. Analysis of /Data Partition File System

We imaged the internal flash memory of the DJI RC, which was composed of partitions, as shown in Table 3. Among these partitions, the user data and drone flight information were located in partition 66, which is the /data partition. Therefore, our primary focus for analysis was the data within this partition. This partition is approximately 3.96 GB (4063 MB) in size and uses the EXT4 file system. The overall structure of the directories and files resembles a typical Android file system.

In Section 6.2, all of the installation files of the apps installed on the DJI RC for analysis are located in the subdirectories of /data/app/ and /data/app-lib/. In Section 6.3, the configuration and log files generated by the apps for analysis are found in the subdirectories of /data/data/ and /data/media/0/Android/data/, respecitively. Additionally, in Section 6.4, the multimedia data for analysis can be located in the subdirectory /DCIM/DJI Album on the external SD card.

### 6.2. Analysis of APK File for Application Behavior

The DJI RC, like a typical Android device, can have various apps installed, and these apps may store user traces (such as account information, usage history, etc.) in their generated settings and log files. Therefore, it is essential to identify the list of all installed apps on the DJI RC and analyze the behavior of each app along with the logs it generates.

As mentioned in Section 5.1.1, we acquired root access to the DJI RC using the DJI RC FCC. This allowed us to execute the pm list packages command through the ADB shell to retrieve a list of all installed apps on the DJI RC. Additionally, the /data/system/packages.xml and /data/system/packages.list files were extracted to obtain information about the installed apps. Furthermore, by examining the imaged /data partition using file system analysis tools like EnCase, FTK Imager, Autopsy [34], etc., we could explore the directory structure created under /data/data/ based on the package names of the apps.

Using these methods, we were able to identify a total of 38 apps installed on the DJI RC. The DJI RC has a customized Android OS, as mentioned in Section 3.1, and it does not have a traditional home screen. Instead, it always launches the DJI Fly app and positions it at the top of the screen. As a result, users might assume that only the DJI Fly app exists on the DJI RC. However, in reality, there are 38 apps whose installation statuses are not visible to the user. Given the potential for these apps to store user information or drone-related data in logs, a comprehensive analysis of them was necessary. Please see Table 5 for the list of identified apps.

To analyze the behavior of the apps, we needed access to the APK (Android Package Kit) files, also known as Android Application Packages. APK files are the installation files for specific apps, and in the Android ecosystem, after installing an app, its APK file is copied to the /data/app/∗ subdirectory. Hence, extracting APK files from this directory for all installed apps on an Android device is possible.

APK files serve as both the installer for an app and contain the app’s executable files, which are DEX files. These DEX files can be statically analyzed using reverse engineering tools like APKtool [35], JADX [36], JEB Decompiler [37], thus allowing us to understand the behavior of the app. Additionally, the SO files, which are libraries used by the app, can be reverse engineered using tools like IDA Pro [38], Ghidra [39], etc. Moreover, for dynamic analysis, network data capture, and hooking into an app’s internal logic, tools like Frida [40] can be employed.

We analyzed all 38 apps, and the results show that 28 of these apps are developed by Google and are part of the Android Opensource Project (AOSP) [41]. Additionally, nine apps are manufactured by DJI, and Qualcomm produces the remaining app.

#### 6.2.1. Apps Developed by Google

We analyzed the 28 apps developed by Google based on the AOSP framework by comparing them to the source code of the original apps available on the AOSP website. As a result, we found that 27 of these apps were originally developed by Google. These apps do not store user information in their logs or settings, so we excluded them from the digital forensics analysis.

**Figure 4:**
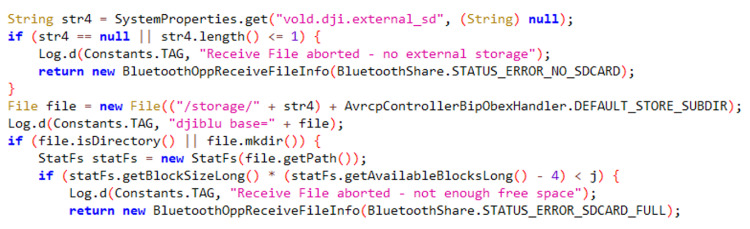
Source code of Bluetooth app on DJI RC.

However, one app, Bluetooth.apk (com.android.bluetooth), stood out as being customized by DJI based on the AOSP framework. This app provides various Bluetooth-related functionalities, including Bluetooth connection management, file transfer, audio streaming, and interaction with peripheral devices. DJI customized this app to handle the synchronization of multimedia files captured by the drone to the DJI RC. The process works as follows: when the DJI RC synchronizes multimedia files from the drone, the files are initially stored on the external SD card. If there is insufficient space on the external SD card, they are stored on the internal flash memory’s SD card. The synchronization process fails if even the internal SD card lacks sufficient storage. Figure 4 represents a portion of the decompiled source code related to this functionality.

#### 6.2.2. App Developed by Qualcomm

This app, listed as number 12 in Table 5, was developed by Qualcomm. It includes the functionality to wirelessly share the display from a smartphone or tablet using Wi-Fi display. It is a modified version of the Android default Gallery app, which allows users to view photos and videos they have captured. This app is typically a default when using devices equipped with Qualcomm chipsets. Since the DJI RC uses the Qualcomm APQ8053 SoCs for IoT chipsets, this app is installed on the device.

#### 6.2.3. Apps Developed by DJI

We conducted a detailed analysis of the following nine apps developed by DJI. In conclusion, among these apps, the two that are crucial for forensic analysis from a digital forensic perspective are the DJI Fly app (dji.go.v5), which is used for operating the DJI RC during flights, and the dpad_setup.apk (com.dpad.setup) app.

***sysobserver.apk (com.dji.sysobserver):*** This app appears to be a debugging tool created by DJI for reference during product development and is not intended to run under normal circumstances. When we forcibly executed the main activity (com.dji.sysobserver.MainActivity) of this app using ADB commands, it generated a systrace button. Clicking this button logs the app’s operation status via logcat and creates a system trace file with a name like atrace_YYYYMMDD_hhmmss.out in the /data/media/0/Android/data/com/dji.sysobserver/files/ directory.

**Figure 5:**
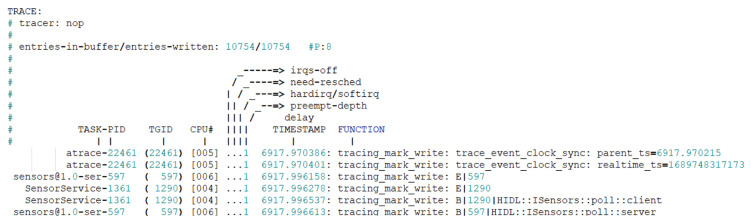
Atrace log contents of Sysobserver App on DJI RC.

This file, as shown in Figure 5, contains information such as process name, process ID, CPU number, execution time, function names, and execution content. It is generated as a result of running a modified Android version of the Linux ftrace that is similar to the atrace tool. However, since this app does not operate under normal circumstances and does not contain user information, it can be excluded from the digital forensic analysis.

***dpad_devicestest.apk (com.dpad.devicetest):*** This app includes features for testing various functions such as the touchscreen, audio, calls, Wi-Fi, camera, and sensors. It appears to be a modification of the default developer testing app provided by Android, with the log paths set to DJI-RC-related directories. Like the previously mentioned apps, this app does not operate under normal circumstances and does not contain user information in the files it generates. Therefore, it can be excluded from the digital forensic analysis.

***dpad_fuli.apk (com.dpad.fuli):*** This app is similar in functionality to the dpad_devicestest.apk app and is designed for DJI RC testing. It can be excluded from the digital forensic analysis.

***base.apk (dji.go.v5):*** This app is the DJI Fly app, which the DJI RC uses for controlling drone flight. It serves as the primary app that runs continuously on the DJI RC, thus providing various functions such as logging into the DJI server, pairing with the drone, controlling drone flight, and downloading multimedia files captured by the drone.

**Figure 6:**
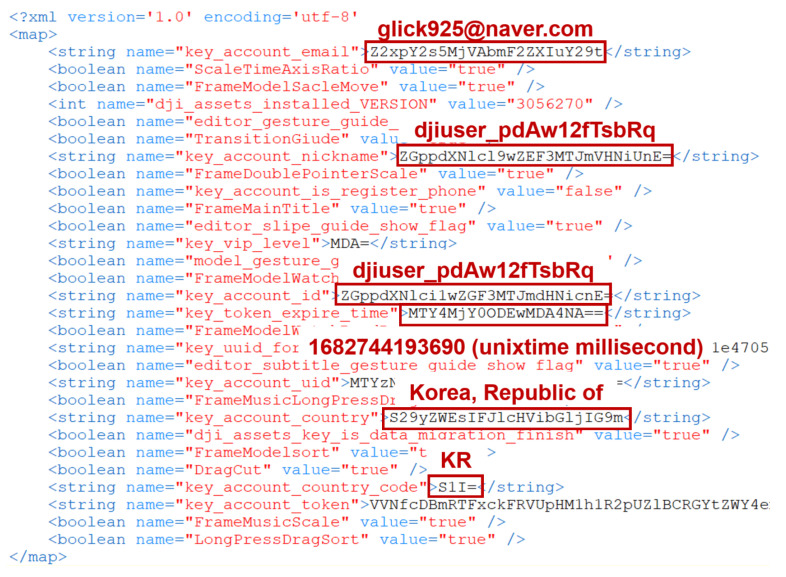
User information in DJI Fly configuration file.

Figure 6 represents the contents of the file located at /data/data/dji.go.v5/shared_prefs/dji.go.v5.xml. Inside this file, various user-related information is stored. For instance, the key_account_email key contains the user’s email, key_account_nickname and key_account_id contain the user’s nickname and ID, respectively, and key_account_country and key_account_country_code store country-related information encoded in Base64. If the user is not logged into the DJI server, this app will only store the key_account_email item.

Additionally, under the path /data/data/dji.go.v5/databases/, we found information related to the pairing of the DJI RC with the drone. Furthermore, in the internal SD card area, specifically at /data/media/0/Android/data/dji.go.v5/files/, we found files containing account information and details of drone flights. It is worth noting that files in this location are not only Base64-encoded, but are also encrypted using a proprietary encryption algorithm.

The numerous log files located under the path /data/media/0/Android/data/dji.go.

v5/files/LOG/CACHE/∗∗ contained a variety of information, including the DJI RC device details, DJI Fly app information, country information where the drone was flown, paired drone information, and location information of the DJI RC during drone flights. However, these files are encrypted on an event basis and then encoded in Base64. To analyze them, reverse engineering of the DJI Fly app was performed to extract the necessary information for decryption.

**Figure 7:**
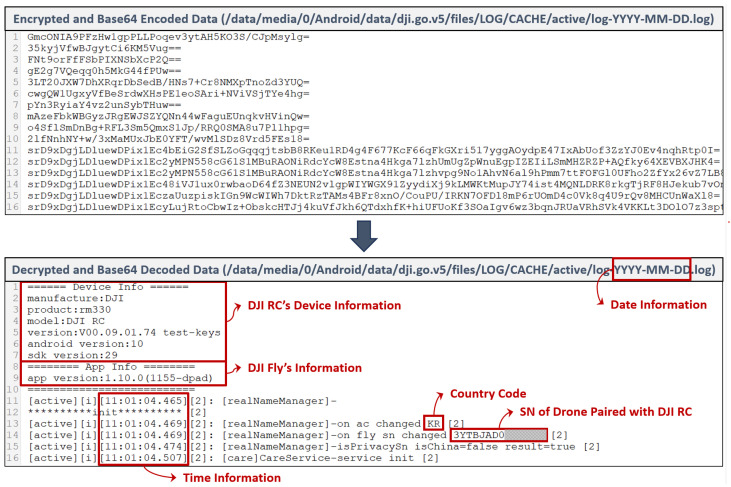
Comparison of data before and after decryption of DJI Fly app log.

The information used to generate the keys required for log decryption and its corresponding results are presented in Table 6. The decryption algorithm is described in Equation (1). An example of decrypting an encrypted log file using this algorithm is provided in Figure 7.

**Theorem** **1.** *Decryption Algorithm for DJI Fly App Logs*

(1) $$\begin{array}{c} C=Base64\_decode(RecordinLogFile) \end{array}$$

(2) $$\begin{array}{c} Key=e9e856d55943731ac585dcda656f95c5 \end{array}$$

(3) $$\begin{array}{c} IV=9d6c5cab5b0281255a222d1c861ddfdf \end{array}$$

(4) $$\begin{array}{c} P=AES\_CBC(C,Key,IV) \end{array}$$

The encrypted file’s contents shown in Figure 7 revealed that each event record is encoded in Base64. To analyze the data, we needed to first decode each record from Base64. After decoding, we could decrypt the data with the obtained decryption key and IV, along with the AES_CBC algorithm. The decrypted results would then appear as shown in the second image.

We found information about the DJI RC and details about the DJI Fly app within the decrypted data. Following that, there were numerous logs along with time information. The time zone for time information is determined based on the country setting of the DJI RC and is not necessarily UTC+0. This file will contain information such as the DJI RC’s country code and the paired drone’s serial number.

All log files under the path /data/media/0/Android/data/dji.go.v5/files/LOG/CACHE/∗ began by recording the Device Info and App Info details, followed by logging their respective data.

***ExtShared.apk (android.ext.share):*** This app is a meaningless dummy app that does not perform any actions. Therefore, it can be excluded from the digital forensic analysis.

**Figure 8:**
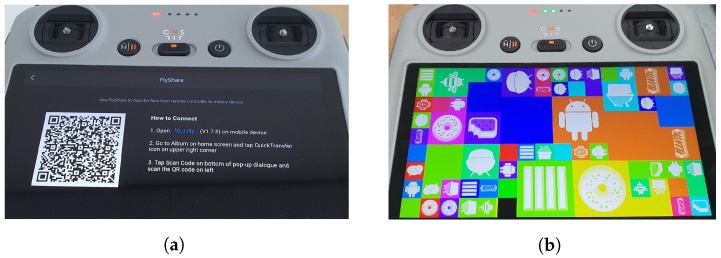
View of dpad_flyshare and dpad_share app. (**a**) dpad_flyshare app. (**b**) dpad_systemui app.

***dpad_flyshare.apk (com.dji.flyshare):*** This app includes a feature that allows for sharing drone flight records with another Android device, not the DJI RC. When the app is launched, a QR code appears as shown in Figure 8a, which can be scanned by another Android device to download flight records. This app can also be excluded from the digital forensic analysis.

***dpad_settings.apk (com.android.settings):*** This app only has the functionality to display information about the DJI RC, such as the model name, CMIIT ID, FCC ID, etc., through a panel. It can also be excluded from the digital forensic analysis.

***dpad_systemui.apk (com.android.systemui):*** This app is simply used for testing the System UI, as shown in Figure 8b. It can also be excluded from the digital forensic analysis.

***dpad_setup.apk (com.dpad.setup):*** This app includes functions related to user account settings such as account activation, login, account recovery, and more. During these processes, it uses WebView to request user permissions, manage user authentication, and handle terms and conditions approval or rejection, as well as manage the initial setup, DJI drone account authentication, login, registration, profile editing, and more.

**Figure 9:**
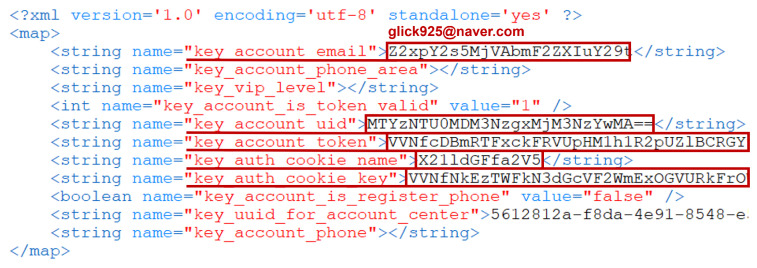
Configureation file os dpad_setup app on DJI RC.

Since this app stores relevant information in internal configuration files during the processing of account information, it should be included as a digital forensic analysis target. Figure 9 shows the content of the /data/data/com.dpad.setup/shared_prefs/com.dpad.setup.xml file, where the user account information is encoded in Base64. This information can be used to identify the account details of users who have used the DJI RC.

### 6.3. Analysis of DJI RC Artifacts (System/App Logs)

Based on the file system analysis and app behavior analysis results, we focused on analyzing critical data from a digital forensic perspective to achieve our research objectives—the 7 Ws:

- We analyzed user account information to determine ’WHO’ operated the drone using DJI RC.
- To find out ’WHICH’ drone flights and ’WHEN’ they were user-controlled, we analyzed information about drones paired with the DJI RC.
- We also analyzed ’WHERE’ the user operated the drone by examining the location information during drone flights.
- We analyzed the flight paths to determine ’WHERE to WHERE’ the drone flew.
- We analyzed multimedia files synchronized from the drone to the DJI RC to understand’ WHAT’ the user captured during drone flights.

#### 6.3.1. Pilot Information

**Figure 10:**
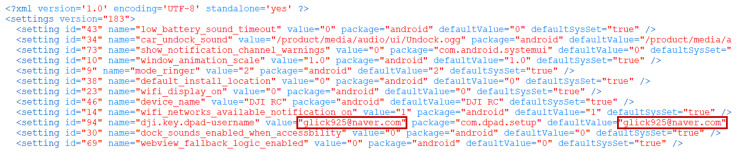
settings.global.xml file containing user account.

The artifacts from the DJI RC that contain information about users who accessed DJI servers through the DJI Fly app are found in four files listed in Table 7. By analyzing these files, we determined ’who’ the person using the DJI RC is (**Q1: WHO**). The dji.go.v5.xml and com.dpad.setup.xml files have already been confirmed, as shown in Figure 6 and Figure 9, respectively. The settings.global.xml file, shown in Figure 10, contains various information related to the settings of each app installed on the DJI RC. This information includes where user-entered account details related to the dji.dpad.setup app are stored.

**Figure 11:**
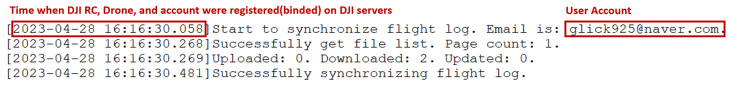
DJIFRSyncLog_YYYY-MM-DD_[HH-MM-SS].txt file containing user account and binding time.

The DJIFRSyncLog_YYYY-MM-DD_[HH-MM-SS].txt file logs the results of registering DJI accounts and drones with DJI servers through the DJI Fly app on the DJI RC. DJI refers to this process as ‘binding’. Therefore, information like that in Figure 11 allows us to determine the user account and also provides the timestamp of when the DJI RC, DJI drone, and DJI account were registered with the DJI server. If we add a new drone binding through the DJI RC, a new file will be generated with the timestamp of that event.

#### 6.3.2. Drone Pairing Information

The artifacts containing information about the drones paired with the DJI RC, as listed in Table 8, provide insights into **Q2. WHICH**—‘which’ drone was paired with the DJI RC and when this pairing occurred.

**Figure 12:**
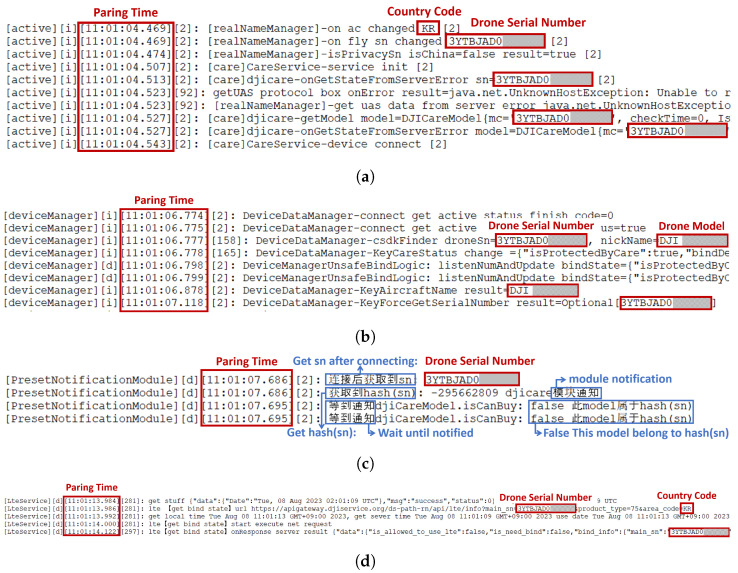
Drone pairing information #1. (**a**) /data/.../active/∗.txt File containing drone pairing information. (**b**) /data/.../deviceManager/∗.txt file containing drone pairing information. (**c**) /data/.../DJINotificationService/∗.txt file containing drone pairing information. (**d**) /data/.../LteService/∗.txt file containing drone pairing information.

The files /data/.../active/∗.txt, /data/.../deviceManager/∗.txt, /data/.../DJINotificationService/∗.txt and /data/.../LteService/∗.txt contain logs of the communication between the DJI RC and the drone when they are paired, including information about the binding of the drone. These file contents are in Figure 12a–d. Analyzing these files can reveal ’which’ drone was paired with the DJI RC (**Q2: WHICH**) and provided information about when the pairing occurred, the serial number and model name of the drone, and the country code set on the DJI RC at the time of pairing.

**Figure 13:**
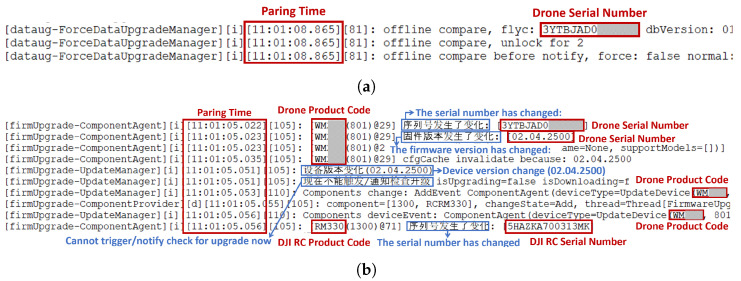
Drone pairing information #2. (**a**) /data/.../UP_DATA_ALL/∗.txt file containing drone pairing information. (**b**) /data/.../UP_NEW_ALL/∗.txt file containing drone pairing information.

The files /data/.../UP_DATA_ALL/∗.txt and /data/.../UP_NEW_ALL/∗.txt contain logs of the process where the DJI RC and the drone pair and retrieve detailed information about the drone. The internal information in these files is shown in Figure 13a,b. Analyzing these files can provide information about the pairing timestamp, the drone’s serial number, firmware version, product code, and the DJI RC’s serial number and product code during pairing.

The /data/data/dji.go.v5/databases/dji.db file contains a table named dji_component_active_model_DJICaredModelV2, and the mc column within this table stores the serial number of the drone paired with the DJI RC. Unlike other files, this one is not encrypted and can be easily analyzed.

#### 6.3.3. Location Information

The data types in the DJI RC that contain location information include the DJI RC’s location and the flown drone’s location. The artifacts that contain such information are listed in Table 9. Analyzing the file containing the DJI RC’s location can reveal when and where the user controlled the drone (**Q3: WHEN** and **Q4: WHERE**), while analyzing the file containing the drone’s location can provide information about where the drone was flown to and from (**Q5: WHERE**).

***DJI RC Location:*** The location information for the DJI RC represents the location of the person controlling the drone’s flight. The artifacts containing this information include /data/.../CACHE/∗.txt, /data/.../CACHE/AeroScopeLogic/∗.txt, and /data/.../CACHE/AttitudeBar/∗.txt.

**Figure 14:**
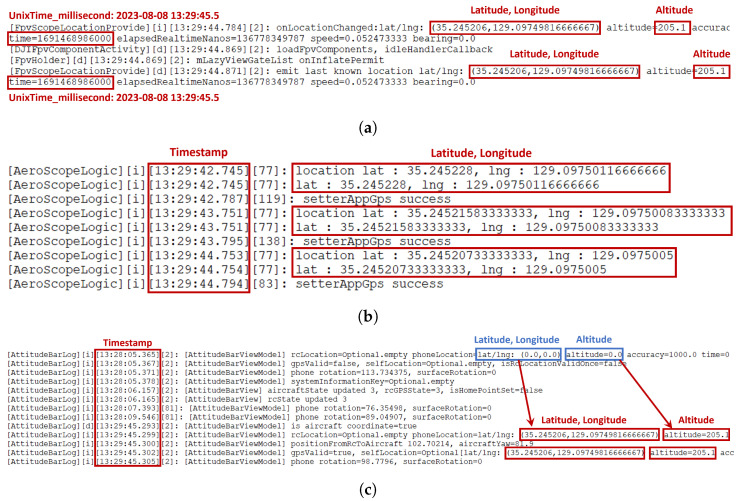
DJI RC location information #1. (**a**) /data/.../CACHE/∗.txt file containing DJI RC location information. (**b**) /data/.../CACHE/AeroScope/∗.txt file containing DJI RC location information. (**c**) /data/.../CACHE/AttitudeBar/∗.txt file containing DJI RC location information.

The CACHE and AeroScopeLogic artifacts only store location information when the DJI RC is connected to the internet via Wi-Fi. However, the Attitude artifact, as shown in Figure 14c, records latitude and longitude as ‘0’ when the internet is not connected, and once the internet connection is established, it records the actual latitude and longitude. This means that until 13:28:05, there was no internet connection and no location information. However, at 13:29:45, when the internet connection was established, it started recording location information.

**Figure 15:**
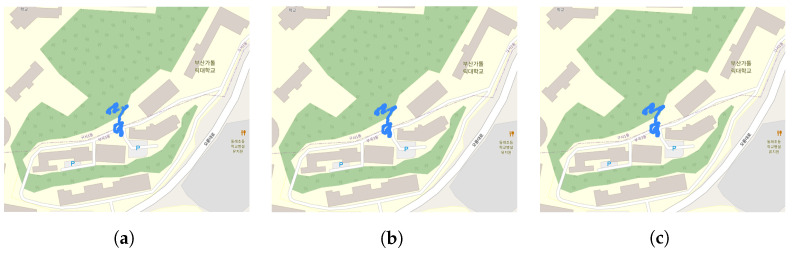
DJI RC location. (**a**) CACHE artifacts. (**b**) AeroScope artifacts. (**c**) AttitudeBar artifacts.

Figure 15 displays the DJI RC’s location information stored in these three artifacts on a map, which is marked in blue. This information can be used to determine if the user traveled to these locations while controlling the drone’s flight.

**Figure 16:**
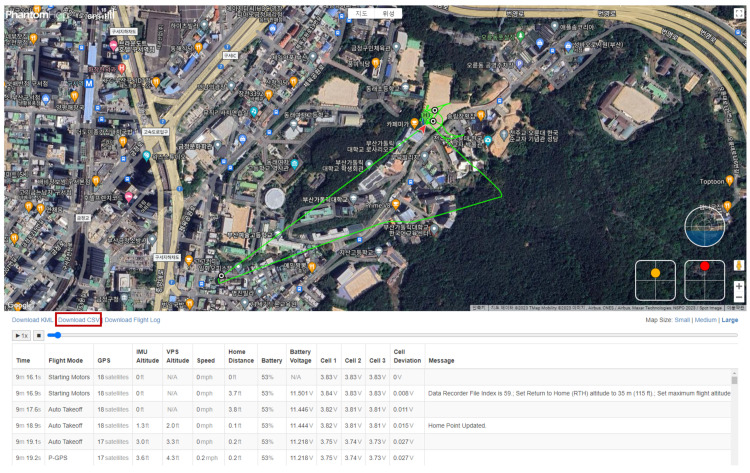
Decrypting the DJIFlightRecord file and viewing the flight records using Phantom Help site.

***Drone Location:*** The DJIFlightRecord_YYYY-MM-DD_[HH-MM-SS].txt file is a drone flight log generated by the DJI Fly app, and it is encrypted. The DJI Flight Log Viewer - Phantom Help [13] website offers a service to decrypt this file, parse the flight records contained within, and display them on a map. Figure 16 shows a screen where flight records can be viewed on this website. We can download the decrypted flight records in the CSV file format by clicking on the Download CSV option below the map.

The CSV file contains a total of 186 columns, and from a digital forensics perspective, the meaningful columns are as listed in Table 10. In essence, this file contains information such as the time when the drone was flown, its location (latitude, longitude, and altitude), flight status, the connection between the DJI RC and the drone, the serial numbers of the cameras mounted on the drone, the drone’s model, and more. In particular, we can determine the drone’s flight path by analyzing the time, location information, and flight status together.

There are four possible flight statuses:

- **Auto Takeoff**: This status indicates the start of the flight.
- **P-GPS**: This status indicates that the DJI RC’s sticks control the drone.
- **Go Home**: This status indicates that the Return To Home (RTH) function is being used, and the drone is returning to its initial takeoff position.
- **Auto Landing**: This status indicates the drone is concluding its flight and landing.

Analyzing this information allows us to understand when the user issued commands to the drone during the flight.

### 6.4. Multimedia Data: Photos and Videos Taken from Drone

While flying the drone and capturing multimedia data, they are initially stored on it. As per the behavior of the Bluetooth.apk (com.android.bluetooth) app, analyzed in Section 6.2.1, the multimedia data stored on the drone are synchronized with the DJI RC via Bluetooth. This data are first saved on DJI RC’s external SD card in a format similar to Table 11. The multimedia data include photos in JPEG format and video files in MP4 format. These files were extracted and analyzed using standard metadata analysis techniques, including examining the EXIF/XMP data within the JPEG files. This analysis helped us determine what the user was capturing while flying the drone (**‘Q5: WHAT’**).

Table 12 demonstrates the parsing of various sensor information from the EXIF/XMP data of such a photo captured by the drone. This data includes information from sensors like the camera, GPS, gyroscope, and more. Analyzing this information enables us to understand where and under what conditions the drone captured the photo.

## 7. Findings and Discussion

Through this research, we conducted an investigation into the DJI RC, the remote controller for various DJI drones, and proposed digital forensic investigation methods. Firstly, in Section 3, we analyzed various features of the DJI RC, including its connectivity with peripheral devices, the types of internal data, and their storage locations.

Table 13 summarizes the data acquisition related to remote controllers in both existing studies and our research. Only 6 of the 11 prior studies collected data from remote controllers. Notably, four of these studies opted for data collection using programs such as DJI Assistant, thus acquiring minimal data primarily focused on flight records. This constrained data scope raises inherent limitations for analyzing drone pilot behavior. The remaining two studies took a different approach by rooting smartphones as remote controllers and conducting forensic imaging of the flash memory. However, despite having imaged the entire dataset, these papers primarily focused on the analysis of drone flight records alone in one study [17], and in the other, they concentrated on the supplementary analysis of drone pairing information [23].

In contrast, we focused our research on remote-controller-specific devices rather than smartphones. We successfully achieved both comprehensive and selective data acquisition from the flash memory. As a result, we could collect encrypted system logs and application installation files (APKs) effectively.

In Section 5, we proposed methods to acquire the internal flash memory of the DJI RC using a customized Android device with root privileges. We also suggested a method for identifying the files stored under the /data partition, which are encrypted during storage, and how to collect these files selectively while the device is live. The failure to selectively collect these files would require additional efforts to decrypt them.

In Section 6, we conducted a detailed analysis of the collected data. Notably, we discovered that, despite the DJI RC lacking a home screen and the ability to install and run other apps, there were 38 different apps present. By reverse engineering all these apps and analyzing their behavior, we identified apps that store essential data from a digital forensic perspective. We also conducted an in-depth analysis of numerous files to identify and analyze various artifacts necessary to achieve the goals of digital forensic investigations, thereby uncovering artifacts suitable for addressing the seven Ws from a digital forensic perspective, specifically user Information, drone pairing information, location information, and multimedia data.

The seven Wss answers to the data we studied are as follows:

- **Q1: Who operated the drone (WHO)?**
  The individual who operated the drone is identified as **glick925@naver.com**, based on analyzing the four artifacts presented in Section 6.3.1.
- **Q2: Which drone was operated (WHICH)?**
  The drone model is a DJI *** **, the serial number is 3YTBJAD0******, and the product code is WM***. This drone was paired with a DJI RC having a product code of RM330 and a serial number of 5HAZKA700313MK, as determined from the analysis of seven artifacts described in Section 6.3.2.
- **Q3: When was the drone operated (WHEN)?**
  The drone was operated at approximately **13:29:45**, as revealed by the analysis of three artifacts in Section 6.3.3.
- **Q4: Where was the drone operated (WHERE)?**
  The location where the user operated the drone is approximately **latitude 35.245206**, **longitude 129.09749816666667**, and **altitude 205.1**, which is in the vicinity shown in Figure 15. This information was obtained from analyzing three artifacts in Section 6.3.3.
- **Q5: From where to where was the drone’s flight controlled (WHERE to WHERE)?**
  The route of the drone’s flight from one location to another is depicted in Figure 16 and corresponds to the information obtained from the analysis of the remaining artifact in Section 6.3.3.
- **Q6: What did the drone capture (WHAT)?**
  The content captured by the drone, **multimedia data (i.e., photos and videos)**, can be determined by examining the files stored in the two paths on the external SD card, as discussed in Section 6.4.

In contrast to previous studies that focused primarily on analyzing drone flight records, limited drone pairing information, and multimedia data captured by the drone, our ability to conduct comprehensive and detailed digital forensics analysis of the drone pilots can be attributed to two primary factors. First and foremost, we collected all the target data from the remote controller, and secondly, we rigorously examined all the collected data. Our analysis, particularly concerning drone-flight-related activities within the DJI Fly app, involved a detailed investigation, including data encryption and decryption, which was achieved through reverse engineering. We meticulously analyzed the system and app logs to maximize the identification of the pilot’s activities.

The limitations of this research are as follows:

***Failure of Data Acquisition:*** The first limitation pertains to the failure to acquire a root privilege to the DJI RC, thus resulting in the inability to collect data from the flash memory. We employed the DJI RC FCC program to root the DJI RC, which exploits vulnerabilities in the Linux kernel or Android OS of the DJI RC to obtain root access. If the firmware of the DJI RC is updated and the existing vulnerabilities are not triggered, we cannot acquire root privileges for the DJI RC. Consequently, flash memory imaging and the selective collection of encrypted files would also fail. Therefore, there is a need for research focused on analyzing vulnerabilities in the DJI RC to develop exploits for obtaining root access.

***Decryption of File System:*** Another limitations is the inability to decrypt the encrypted directories and files identified in Table 4. This encryption is presumed to be implemented through Android’s file-based encryption (FBE) or DJI’s proprietary program. Resolving this issue would enable the selective extraction and decryption of encrypted files during data collection, thereby eliminating the need to collect specific files in a live state.

***Decryption of DJI Flight Records:*** Another limitation is the inability to decrypt the DJIFlightRecord_YYYY-MM-DD_[HH-MM-SS].txt files used for analyzing the drone locations in Section 6.3.3. These files are decrypted by the DJI Fly app, as mentioned in Section 6.2.3. Furthermore, reverse engineering of the behavior of dji.go.v5 may be necessary to discover the decryption method for these files, thus potentially eliminating the need for external services like DJI Flight Log Viewer - Phantom Help. Furthermore, if the DJI Fly app undergoes updates, the 1 algorithm we reverse engineered may change. In such a case, it could render the decryption of the critical log files pivotal for pilot analysis impossible. Hence, it is essential to monitor the status of DJI Fly app updates consistently.

## 8. Conclusions and Future Work

In recent years, the use of drones has significantly increased worldwide. Drones are generally used positively across various industries. However, there is a growing need for digital forensics investigations into drone systems for cases involving malicious intent, criminal activities, or other potential security concerns. Our research focuses on the DJI RC (remote controller), which is a widely used remote control device for DJI drones, which holds a significant market share globally.

We proposed a data collection methodology from a digital forensics perspective for the DJI RC, as well as selected and analyzed crucial artifacts for uncovering potential criminal activities. Using the analysis methods we have suggested, it is possible to identify the individuals who operated drones using the DJI RC, determine when and where drone flights occurred, and gain insights into the content captured during those flights. Additionally, this methodology can be applied to other DJI remote controllers that use the DJI Fly app, thus making it not limited to the DJI RC alone.

Our future research will focus on tackling the encrypted data within the DJI RC. Specifically, we plan to research methods for decrypting the encrypted data in the file system, along with in-depth reverse engineering of the DJI Fly app, to understand how it decrypts files like the DJIFlightRecord_YYYY-MM-DD_[HH-MM-SS].txt, which contain drone location information. For this purpose, the application of machine learning and deep-learning-based encryption-breaking techniques is expected to be explored. Additionally, we aim to conduct vulnerability analyses of drone systems from a security perspective, not just digital forensics, to enhance the overall security of drone systems.

## Figures and Tables

**Table 1:** Related paper list.

No	Year	Ref.	Target Device			Data Acquisition				Data Analysis	
			Drone	Remote Controller	Device Description	Internal Flash Memory	Internal SD Card	External SD Card	Used Tool	Data Description	Used Tool
1	2017	Devon R et al. [16]	O	-	- Drone: DJI Phantom 3	-	O	-	- DJI Assistant	- Drone: Drone Flight Log (DAT File)	DROP
2	2017	Barton et al. [17]	O	O (Smart phone)	- Drone: DJI Phantom 3 Professional, Parrot AR. Drone 2.0 - RC: Motorola G3	O	O	O	- DD and ADB with Rooting : Motorola G3 - DD and Shell without Rooting : Parrot AR. Drone 2.0 - DJI Assistant	- Drone: Drone Flight Log (DAT File), Multimedia Data, Physical Image (Parrot Drone)	CsvView/DatCon
3	2019	Maryam et al. [18]	O	O (Smart phone)	- Drone: DJI Mavic Air, - RC: DJI Mavic Air Remote Controller (with iPhone 6)	-	O	O	- DJI Assistant - iTunes Backup	- Drone: Drone Flight Log (DAT File), Multimedia Data (Image File), - RC (Smartphone): Drone Flight Log (TXT File), App Config Data	CsvView/DatCon, FTK Imager, Autopsy
4	2019	Ankit et al. [19]	O	O (Smart phone)	- Drone: Yuneec Typhoon H, DJI Phantom 4 - RC: Unknown Android Smartphone	-	O	O	- DJI Assistant	- Drone: Drone Flight Log (DAT File, CSV File) - RC (Smartphone): Drone Flight Log (TXT File)	
5	2020	Maryam et al. [20]	O	O (Smart phone)	- Drone: DJI Mavic 2 pro, Mavic air, Spark, Phantom 4 - RC: iPhone 6S	-	O	O	- DJI Assistant - iTunes Backup	- Drone: Drone Flight Log (DAT File), Multimedia Data (Image File, Video File) - RC (Smartphone): Drone Flight Log (TXT File), Drone Paring Information (iTunes Backup)	CsvVew/DatCon, ExtractDJI, FTK Imager, Autopsy
6	2021	Fahad E et al. [21]	O	-	- Drone: DJI Matrice 210, DJI Phantom 4	O	O	O	- Chip-Off : DJI Matric 210, DJI Phantom 4 - DJI Assistant	- Drone: Drone Flight Log (DAT File), Multimedia Data (Image File, Video File), Drone Information (from Drone Chip-Off Internal Flash Memory)	CsvView/DatCon, ExifTool, Magnet AXIOM, Cellebrite Reader, Autopsy
7	2021	Fahad E et al. [22]	O	-	- Drone: VTI Phoenix, DJI Matrice 210	-	O	O	- DJI Assistant	- Drone: Drone Flight Log (DAT File)	CsvView/DatCon
8	2021	Miloš et al. [23]	O	O (Smart phone)	- Drone: DJI Mini 2 - RC: DJI RC-N1 with iPhone7, Samsung Galaxy S7	O	O	O	- DD and ADB with Rooting : Samsung Galaxy S7 - DJI Assistant - Cellebrite Memory Card Reader	- Drone, RC: Multimedia Data (Image File, Video File) - RC (Smartphone): Drone Flight Log (DAT File, TXT File), Drone Paring Artifacts (from Smartphone Data)	Magnet AXIOM, ExifTool, Autopsy
9	2022	Hudan et al. [24]	O	-	Uses Open Dataset (DJI Phantom 3, DJI Phantom 4, DJI Phantom 4 Pro, DJI Inspire 1, DJI Inspire 2, DJI Mavic Pro, DJI Mavic Air)	-	-	-	- Uses Open Dataset (CFReDS Drone Data Set)	- CFReDS Data set: Drone Image (timestamp)	DroneTimeline
10	2022	Lee et al. [25]	O	O (Smart phone)	- Drone: DJI Phantom 3, DJI Mavic Mini, DJI Mini 2 - RC (iPhone 7, Samsung Galaxy S7)		O	O	- DJI Assistant	- Drone: Drone information (from External SDcard) - Drone, RC: Multimedia Data (Image File, Video File) - RC (Smartphone): Drone Flight Log (TXT File)	CsvView/DatCon, Airdata UAV
11	2023	Vikas et al. [26]	O	-	Uses Open Dataset (DJI Mavic Air 2S, DJI Mavic Pro, DJI Mavic Pro 2, DJI Inspire 2, DJI Mavic Mini, DJI Phantom 4, Parrot Disco)	-	-	-	- Uses Open Dataset	- Proposed the Drone Forensic Framework	
12	2023	Our Study	-	O (RC Device)	RC: DJI RC	O	O	O	- DD and ADB with Rooting - FTK Imager	- RC: Drone Flight Log (DAT File, TXT File), Multimedia Data (Image File, Video File), Pilot Information, Drone Pairing Information	

**Table 2:** Specifications of of DJI RC.

Item	Description
Model Name	DJI RC
Product Name	rm330
Application Processor	Qualcomm APQ8053 SoCs for IoT
Firmware Version	V01.03.0500
Operating Systems	Android 10 (Customized)
SDK Version	29
Drone Controller App	DJI Fly (dji.go.v5)
Internal Flash Memory	8 GB (Samsung KMFN60012B)
External SD Card	64 GB (Samsung EVO Plus)
Supported Drone	DJI Mini 3 Pro, Mavic 3, Air 2S, Mavic 3 Classic, Mini 3, Mavic 3 Pro

**Table 3:** Partition information of DJI RC’s internal flash memory.

No	Partition Name	Format	No	Partition Name	Format	No	Partition Name	Format
1	Unallocated	-	24	Unallocated	-	47	mota	Unknown
2	modem	FAT16	25	aboot	ELF	48	dip	Unknown
3	Unallocated	-	26	abootbak	ELF	49	mdtp	Unknown
4	fsc	Unknown	27	dtbo	Unknown	50	syscfg	Unknown
5	Unallocated	-	28	dtbobak	Unknown	51	mcfg	Unknown
6	ssd	Unknown	29	vbmeta	Unknown	52	Unallocated	-
7	sbl1	ELF	30	vbmetabak	Unknown	53	lksecapp	ELF
8	sbl1bak	ELF	31	boot	Kernel Image	54	lksecappbak	ELF
9	rpm	ELF	32	recovery	Kernel Image	55	cmnlib	ELF
10	rpmbak	ELF	33	devinfo	Unknown	56	cmnlibbak	ELF
11	tz	ELF	34	system	EXT4	57	cmnlib64	ELF
12	tzbak	ELF	35	vendor	Unknown	58	cmnlib64bak	ELF
13	devcfg	ELF	36	Unallocated	-	59	keymaster	ELF
14	devcfgbak	ELF	37	cache	EXT4	60	keymasterbak	ELF
15	dsp	EXT4	38	persist	EXT4	61	Unallocated	-
16	modemst1	Unknown	39	misc	Unknown	62	apdp	Unknown
17	modemst2	Unknown	40	keystore	Unknown	63	msadp	Unknown
18	Unallocated	-	41	config	Unknown	64	dpo	Unknown
19	DDR	Unknown	42	dji_persist	EXT4	65	blackbox	EXT4
20	fsg	Unknown	43	djita	ELF	66	data	EXT4
21	sec	Unknown	44	Unallocated	-	67	Unallocated	-
22	Unallocated	-	45	limits	Unknown			
23	splash	Unknown	46	Unallocated	-			

**Table 4:** Information of/data partition sub-directory.

Directory Name	Encryption	Description	Directory Name	Encryption	Description
adb	-		misc_ce	Encrypted	
anr	Encrypted		misc_de	Encrypted	
apex	-		nfc	Encrypted	
app	Encrypted	APK Files	ota	-	
app-asec	-		ota_package	-	
app-ephemeral	-		per_boot	-	
app-lib	Encrypted	APK Native Libraries	preloads	-	
app-private	-		property	Encrypted	
app-staging	-		resource-cache	-	
backup	-		rollback	-	
bootchart	-		rollback-observer	-	
cache	Encrypted		server_configurable_flags	-	
dalvik-cache	Encrypted		ss	-	
data	-	App Config and Logs	system	Encrypted	System Config and Logs
dpm	-		system_ce	Encrypted	
drm	-		system_de	Encrypted	
fota	-		tombstones	Encrypted	
gsi	-		unencrypted	-	
hostapd	-		user	-	
local	Encrypted		user_de	Encrypted	
lost+found	-		vendor	Encrypted	
media	-	Internal SD Card	vendor_ce	Encrypted	
mediadrm	-		vendor_de	Encrypted	
misc	Encrypted	Device Config Files			

**Table 5:** List of installed applications on DJI RC.

No	APK Name	APK File Path	Package Name	Developer
1	sysobserver.apk	/system/product/priv-app/sysobserver/	com.dji.sysobserver	DJI
2	TelephonyProvider.apk	/system/priv-app/TelephonProvider/	com.android.providers.telephony	Google
3	MediaProvider.apk	/system/priv-app/MediaProvider/	com.android.providers.media	Google
4	DocumentsUI.apk	/system/priv-app/DocumentsUI/	com.android.documentsui	Google
5	ExternalStorageProvider.apk	/system/priv-app/ExternalStorageProvider/	com.android.externalstorage	Google
6	DownloadProvider.apk	/system/priv-app/DownloadProvider/	com.android.providers.downloads	Google
7	InProcessNetworkStack.apk	/system/priv-app/InProcessNetworkStack/	com.android.networkstack.inprocess	Google
8	DownloadProviderUi.apk	/system/priv-app/DownloadProviderUi/	com.android.providers.downloads.ui	Google
9	ModuleMetadata.apk	/system/product/app/ModuleMetadata/	com.android.modulemetadata	Google
10	CertInstaller.apk	/system/app/CertInstaller/	com.android.certinstaller	Google
11	framework-res.apk	/system/framework/	-	Google
12	SnapdragonGallery.apk	/system/priv-app/SnapdragonGallery/	org.codeaurora.gallery	Qualcomm
13	WfdService.apk	/system/priv-app/WfdService/	com.qualcomm.wfd.service	Google
14	OsuLogin.apk	/system/app/OsuLogin/	com.android.hotspot2	Google
15	dpad_devicestest.apk	/vendor/app/dpad_devicestest/	com.dpad.devicetest	DJI
16	SettingsProvider.apk	/system/priv-app/SettingsProvider/	com.android.providers.settings	Google
17	SharedStorageBackup.apk	/system/priv-app/SharedStorageBackup/	com.android.sharedstoragebackup	Google
18	dpad_fuli.apk	/vendor/app/dpad_fuli/	com.dpad.fuli	DJI
19	base.apk	/data/app/dji.go.v5-ShCjxx-gV0lw5hlV8s1O-A==/	dji.go.v5	DJI
20	webview.apk	/system/product/app/webview/	com.android.webview	Google
21	InputDevices.apk	/system/priv-app/InputDevices/	com.android.inputdevices	Google
22	ExtShared.apk	/system/app/ExtShared/	android.ext.shared	DJI
23	Telecom.apk	/system/priv-app/Telecom/	com.android.server.telecom	Google
24	ExtServices.apk	/system/priv-app/ExtServices/	android.ext.services	Google
25	PackageInstaller.apk	/system/priv-app/PackageInstaller/	com.android.packageinstaller	Google
26	dpad_flyshare.apk	/vendor/app/dpad_flyshare/	com.dji.flyshare	DJI
27	StorageManager.apk	/system/product/priv-app/StorageManager/	com.android.storagemanager	Google
28	dpad_settings.apk	/vendor/app/dpad_settings/	com.android.settings	DJI
29	PlatformNetworkPermissionConfig.apk	/system/priv-app/PlatformNetworkPermissionConfig/	com.android.networkstack.permissionconfig	Google
30	TeleService.apk	/system/priv-app/TeleService/	com.android.phone	Google
31	Shell.apk	/system/priv-app/Shell/	com.android.shell	Google
32	FusedLocation.apk	/system/priv-app/FusedLocation/	com.android.location.fused	Google
33	dpad_systemui.apk	/system/product/priv-app/dpad_systemui/	com.android.systemui	DJI
34	PermissionController.apk	/system/priv-app/PermissionController/	com.android.permissioncontroller	Google
35	dpad_setup.apk	/system/product/priv-app/dpad_setup/	com.dpad.setup	DJI
36	Bluetooth.apk	/system/app/Bluetooth/	com.android.bluetooth	Google
37	PlatformCaptivePortalLogin.apk	/system/app/PlatformCaptivePortalLogin/	com.android.captiveportallogin	Google
38	gboard_go.apk	/vendor/app/gboard_go/	com.google.android.inputmethod.latin	Google

**Table 6:** Key generation information for decrypting log of DJI Fly app.

Encryption Information	Value
Key Generation Algorithm	PBKDF2WithHmackSHA1
Passphrase	DJILog@SimpleEncryption
Character Encoding Type	UTF-8
Key Size	128bits
Iterations	1000
Salt	c8570ac98cc615aa6a6b97b3f20f1b41
Generated Decryption Key	e9e856d55943731ac585dcda656f95c5

**Table 7:** Artifacts list containing user information.

Directory Path	File Name	Protection	Description
/data/data/dji.go.v5/Shared_prefs	dji.go.v5.xml	Base64	Email Address, Nickname, Registerd Country
/data/data/com.dpad.setup/shared_prefs	com.dpad.setup.xml	Base64	Email Address
/data/system/users/0	settings_global.xml	-	Email Address
/data/media/0/Android/data/dji.go.v5/files/ FlightRecord/SyncResult	DJIFRSyncLog_YYYY -MM-DD_[HH-MM-SS].txt	-	Email Address

**Table 8:** Artifacts list containing drone pairing information.

Directory Path	File Name	Protection	Description
/data/media/0/Android/data/dji.go.v5/files/ LOG/CACHE/active	log-YYYY-MM-DD.log	Encryption, Base64	Paring Time, Drone SN, Country Code
/data/media/0/Android/data/dji.go.v5/files/ LOG/CACHE/deviceManager	log-YYYY-MM-DD.log	Encryption, Base64	Paring Time, Drone SN, Drone Model
/data/media/0/Android/data/dji.go.v5/files/ LOG/CACHE/DJINotificationService	log-YYYY-MM-DD.log	Encryption, Base64	Paring Time, Drone SN
/data/media/0/Android/data/dji.go.v5/files/ LOG/CACHE/LteService	log-YYYY-MM-DD.log	Encryption, Base64	Paring Time, Drone SN, Country Code
/data/media/0/Android/data/dji.go.v5/files/ LOG/CACHE/UP_DATA_ALL	log-YYYY-MM-DD.log	Encryption, Base64	Paring Time, Drone SN
/data/media/0/Android/data/dji.go.v5/files/ LOG/CACHE/UP_NEW_ALL	log-YYYY-MM-DD.log	Encryption, Base64	Paring Time, Drone SN, Drone Firmware Version Drone Product Code, DJI RC SN, DJI RC Product Code
/data/data/dji.go.v5/databases	dji.db (table: dji_component_ active_model_DJICaredModelV2)	-	Drone SN

**Table 9:** Artifacts list containing DJI RC and drone location information.

Directory Path	File Name	Protection	Description
/data/media/0/Android/data/dji.go.v5/ files/LOG/CACHE	log-YYYY-MM-DD.log	Encryption, Base64	Timestamp(UnixTime millisecond), DJI RC’s Latitude, Longitude, Altitude (Only when DJI RC is Online)
/data/media/0/Android/data/dji.go.v5/ files/LOG/CACHE/AeroScopeLogic	log-YYYY-MM-DD.log	Encryption, Base64	Timestamp(DateTime), DJI RC’s Latitude, Longitude (Only when DJI RC is Online)
/data/media/0/Android/data/dji.go.v5/ files/LOG/CACHE/AttitudeBar	log-YYYY-MM-DD.log	Encryption, Base64	Timestamp(DateTime), DJI RC’s Latitude, Longitude, Altitude
/data/media/0/Android/data/dji.go.v5/ files/FlightRecord	DJIFlightRecord_YYYY -DD-MM_[HH:MM:SS].txt	Encryption	Timestamp(DateTime), Drone’s Latitude, Longitude, Altitude, Flight State, Drone Model, Drone SN, Camera SN of Drone, DJI RC SN

**Table 10:** Important columns in the decrypted flight record CSV file.

Item	Description
CUSTOM.date	Flight Date (M/D/YYYY)
CUSTOM.updatetime	Flight Time (HH:MM:SS.ss)
OSD.latitude	Latitude of Drone
OSD.longitude	Longitude of Drone
OSD.altitude	Altitude of Drone
OSD.flycState	Flight State of Drone
DETAILS.aircraftName	Drone Model
DETAILS.aircraftSerial	Drone SN
DETAILS.cameraSerial	Camera SN of Drone
DETAILS.rcSerial	DJI RC SN

**Table 11:** Multimedia information on external SD card.

Directory Path	File Name	Description
/DCIM/DJI Album/	dji_fly_YYYYMMDD_HHMMMSS_Number_UnixTime(millisecond)_photo.jpg	Photo
/DCIM/DJI Album/	dji_fly_YYYYMMDD_HHMMMSS_Number_UnixTime(millisecond)_video.jpg	Video

**Table 12:** EXIF/XMP data of photos containing information from the drone’s sensors.

Item	Value	Item	Value
ExposureTime	1/2000	ISO	100
ExposureProgram	Program AE	LensInfo	22.4 mm f/2.8
ExposureMode	Auto	GPSAltitude	173.2 m Above Sea Level
FNumber	2.8	GPS Latitude	35 deg 14${}^{\prime }$ 42.06${}^{\prime \prime }$ N
ShutterSpeedValue	1/2000	GPS Longitude	129 deg 5${}^{\prime }$ 51.45${}^{\prime \prime }$ E
MeteringMode	Average	GimbalRollDegree	+0.00
LightSource	Daylight	GimbalYawDegree	−143.4
LightValue	13.9	GimbalPitchDegree	+0.00
Flash	No Flash	FlightRollDegree	−1.7
FocalLength	8.4 mm	FlightYawDegree	−156.6
HyperfocalDistance	2.19 m	FlightPitchDegree	+9.50

**Table 13:** Summary of data acquisition from remote controllers among related studies.

No	Ref.	Remote Controller		Flash Memory Full Imaging Acquisition		Selective Acquisition for Encrypted Files inside Flash Memory	Full Imaging or Seletive File Acquisiton inside SD Card	Partial Data Acquisition inside Flash Memory Using Manufacturer Tool
		**Smartphone**	**RC Device**	**Rooting**	**Chip-Off**			
1	Devon R et al. [16]	-	-	-	-	-	-	-
2	Barton et al. [17]	O	-	O	-	-	O	O (DJI Assistant)
3	Maryam et al. [18]	O	-	-	-	-	O	O (DJI Assistant, iTunes)
4	Ankit et al. [19]	O	-	-	-	-	O	O (DJI Assistant)
5	Maryam et al. [20]	O	-	-	-	-	O	O (DJI Assistant, iTunes)
6	Fahad E et al. [21]	-	-	-	-	-	-	-
7	Fahad E et al. [22]	-	-	-	-	-	-	-
8	Miloš et al. [23]	O	-	O	-	-	O	O (DJI Assistant)
9	Hudan et al. [24]	-	-	-	-	-	-	-
10	Lee et al. [25]	O	-	-	-	-	O	O (DJI Assistant)
11	Vikas et al. [26]	-	-	-	-	-	-	-
12	Our Study	-	O	O	-	O	O	-

## Data Availability

Not applicable.
